# Advancements, challenges, and future perspectives in developing feline herpesvirus 1 as a vaccine vector

**DOI:** 10.3389/fimmu.2024.1445387

**Published:** 2024-09-12

**Authors:** Xinru Luo, Ruiying Liang, Lin Liang, Aoxing Tang, Shaohua Hou, Jiabo Ding, Zibin Li, Xinming Tang

**Affiliations:** ^1^ Key Laboratory of Animal Biosafety Risk Prevention and Control (North) and Key Laboratory of Veterinary Biological Products and Chemical Drugs of Ministry of Agriculture and Rural Affairs (MARA), Institute of Animal Sciences, Chinese Academy of Agricultural Sciences, Beijing, China; ^2^ Shanghai Veterinary Institute, Chinese Academy of Agricultural Sciences, Shanghai, China; ^3^ College of Life and Health, Dalian University, Dalian, China

**Keywords:** feline herpesvirus 1, vaccine vector, CRISPR/Cas9, protective immunity, infectious and zoonotic diseases

## Abstract

As the most prevalent companion animal, cats are threatened by numerous infectious diseases and carry zoonotic pathogens such as *Toxoplasma gondii* and *Bartonella henselae*, which are the primary causes of human toxoplasmosis and cat-scratch disease. Vaccines play a crucial role in preventing and controlling the spread of diseases in both humans and animals. Currently, there are only three core vaccines available to prevent feline panleukopenia, feline herpesvirus, and feline calicivirus infections, with few vaccines available for other significant feline infectious and zoonotic diseases. Feline herpesvirus, a major component of the core vaccine, offers several advantages and a stable genetic manipulation platform, making it an ideal model for vaccine vector development to prevent and control feline infectious diseases. This paper reviews the technologies involved in the research and development of the feline herpesvirus vaccine vector, including homologous recombination, CRISPR/Cas9, and bacterial artificial chromosomes. It also examines the design and effectiveness of expressing antigens of other pathogens using the feline herpesvirus as a vaccine vector. Additionally, the paper analyzes existing technical bottlenecks and challenges, providing an outlook on its application prospects. The aim of this review is to provide a scientific basis for the research and development of feline herpesvirus as a vaccine vector and to offer new ideas for the prevention and control of significant feline infectious and zoonotic diseases.

## 1 Introduction

Owning companion animals has become increasingly popular for many families, and the bond between these animals and humans continues to strengthen. These animals hold significant cultural and emotional value within families, contributing to overall happiness (1–3). Companion animals include a wide variety of species, such as dogs, cats, birds, snakes, and rodents, with dogs and cats being the most common. In China, the number of pet dogs and cats surpassed 100 million in 2020 and has been rising. By 2021, the number of pet cats exceeded that of dogs, making cats the most popular companion animal. As the leading companion animal, cats face numerous threats from infectious diseases and carry zoonotic pathogens. Feline infectious diseases have a profound global impact, threatening the health of domestic cats and posing significant public health concerns due to their potential as carriers of zoonotic pathogens (4). Diseases like feline parvovirus (FPV) and feline calicivirus (FCV) contribute to high morbidity and mortality rates in cats worldwide, while zoonotic infections such as toxoplasmosis, rabies and cat-scratch disease pose health risks to humans (5). Therefore, controlling major feline infectious and zoonotic diseases is crucial not only for maintaining animal health and welfare but also for preventing zoonotic disease transmission, which has significant public health implications.

Vaccination is the most cost-effective and efficient means of disease control. However, research on cat vaccines lags significantly behind that of other animals due to the challenges of managing cat populations, conducting large-scale animal experiments, and the lack of model animals. Currently, the international market offers only the Feline Rhinotracheitis-Calici-Panleukopenia Vaccine (inactivated or attenuated) as core vaccines for feline panleukopenia, viral rhinotracheitis, and calicivirus infections. A few non-core vaccines are available for other diseases, while many vaccines for other significant feline infectious and zoonotic diseases remain in the research stage (6).

Depending on their components, production processes, and delivery vectors, vaccines are generally categorized into live/attenuated vaccines, inactivated vaccines, toxoid vaccines, subunit vaccines, live vector vaccines, and nucleic acid vaccines. Live vector vaccines are created by genetically engineering microorganisms such as viruses, bacteria, or parasites to weaken or eliminate their pathogenicity while carrying one or several protective antigens of the pathogen (7). These vaccines utilize the host’s infection capability to deliver and present the antigen targets, offering broad immune responses and eliminating the need for adjuvants, thus having excellent application prospects (8–12).

Research on viruses as vaccine vectors is the most advanced, with several commercial viral vector vaccines already available. Examples include vaccines using poxviruses (13), adenoviruses (14), parvoviruses (15), Newcastle disease viruses (16), and herpesviruses (17) as vectors. Feline herpesvirus type 1 (FHV-1) is an enveloped dsDNA virus in the Herpesviridae family. FHV-1 infection causes feline viral rhinotracheitis (FVR), accounting for over half of viral upper respiratory infections in cats (18). FHV-1 has several unique advantages as a vaccine vector: (1) Its double-stranded DNA structure ensures stable genetic information inheritance. (2) Among many pathogens that can infect cats, FHV-1 has low pathogenicity and high safety. Genetic manipulation can knock out multiple virulence-related genes to further enhance its safety as a vaccine vector.

Developing multivalent vaccines based on FHV-1 offers the advantage of “one shot for multiple diseases,” reducing the number of immunizations and stress on animals, thereby improving animal welfare. This approach has significant production and application value. This paper reviews the genome and biological characteristics of FHV-1 and its research as a vaccine vector, including the immune response and protective effects of expressing important antigens of significant feline and zoonotic diseases. It also analyzes the technical bottlenecks and challenges faced, and prospects its application, aiming to provide a scientific basis for the development of FHV-1 as a vaccine vector and offer new ideas for controlling significant feline infectious and zoonotic diseases.

## 2 Feline herpesvirus: structural characteristics and advantages as a vaccine vector

**Figure 1:**
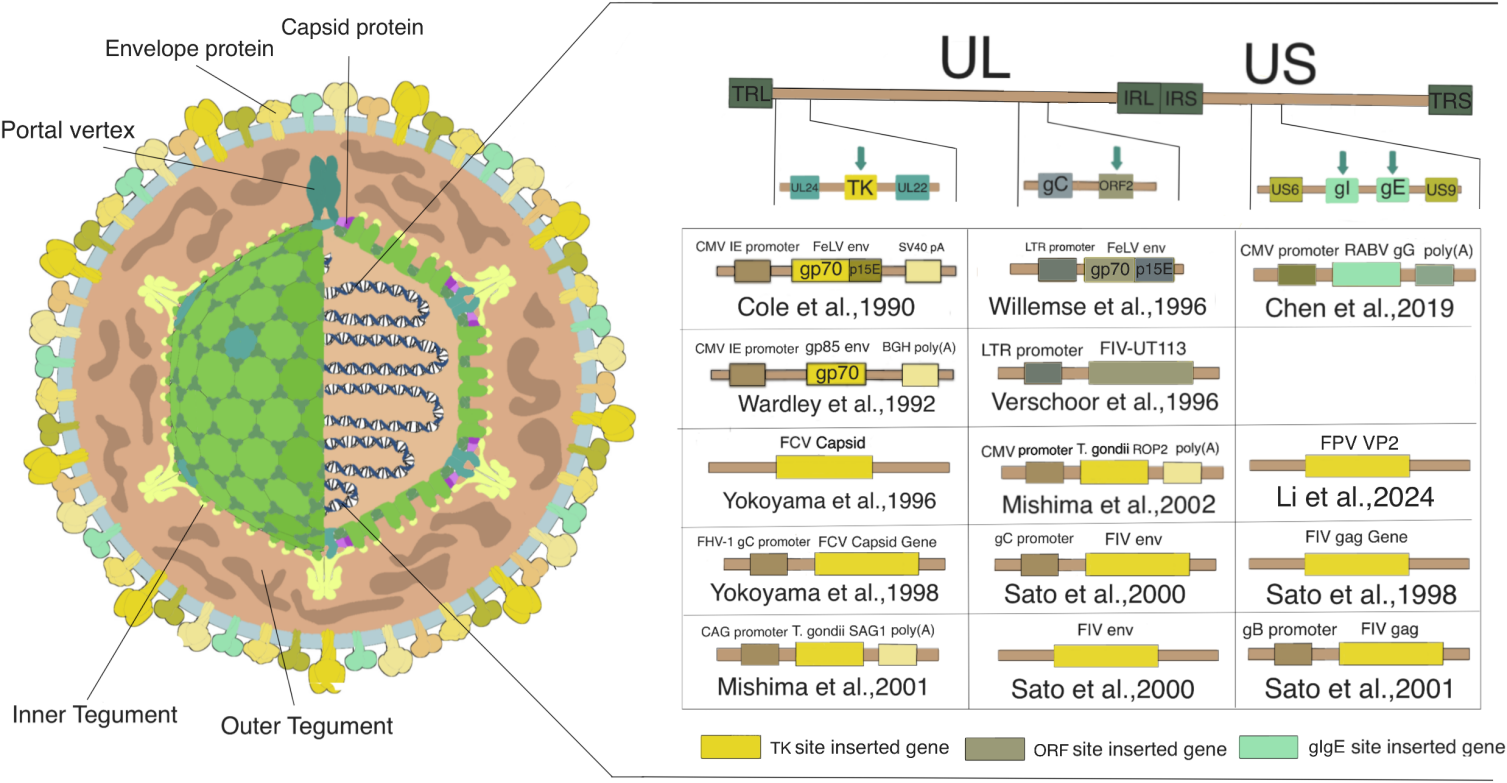
Structure of feline herpesvirus and genetic modification sites for vaccine development. The structure of the Feline Herpesvirus (FHV-1) particle, highlighting key components such as the envelope protein, capsid protein, portal vertex, inner and outer tegument. The genomic map of FHV-1 shows the unique long (UL) and unique short (US) regions, along with the terminal repeat sequences (TRL, TRS) and internal repeat sequences (IRL, IRS). Various genetic modification sites used for inserting genes into the FHV-1 genome for recombinant vaccine development are highlighted: TK (Thymidine Kinase) site: Genes inserted at the TK site are represented by yellow boxes. ORF2 (Open Reading Frame 2) site: Genes inserted at the ORF2 site are represented by brown boxes. gC (glycoprotein C) site: Genes inserted at the gC site are represented by green boxes. gI/gE (glycoprotein I and E) sites: Genes inserted at the gI and gE sites are represented by light brown boxes. The figure lists studies that utilized these insertion sites to express various target antigens for vaccine development. These modifications facilitate the construction of effective recombinant vaccines against different feline pathogens, leveraging the FHV-1 vector.

Feline herpesvirus (FHV-1) is a double-stranded DNA virus of the genus Varicellovirus in the subfamily Alphaherpesvirinae. The genome includes 23 virus-associated proteins and 13 types of glycoproteins, eight of which (gB, gC, gD, gE, gG, gH, gI, and gL) are crucial for regulating FHV-1 replication and infection (19–21). The diameter of FHV-1 virions ranges from 120 nm to 180 nm, varying with the infected tissue and the maturation state of the virus particles. The FHV-1 structure primarily consists of nucleic acid, capsid, tegument, and envelope. The genome length of the feline herpes virus is about 136 kb, with a G-C content of 45%-50%. It is divided into four parts: the unique long (UL) region, the unique short (US) region, the terminal repeat sequences (TRS) at both ends of the US region, and the internal repeat sequences (IRS). The capsid is composed of 150 hexagonal and 12 pentagonal structures arranged radially, ensuring the stability of the genomic DNA (**Figure 1**). The tegument, an irregular protein matrix layer, can be observed under an electron microscope. The outermost layer is the envelope, featuring radially arranged spikes that are essentially glycoproteins (22).

The genome of FHV-1 contains numerous essential and non-essential genes, with the non-essential genes being replaceable by exogenous sequences that encode immunogenic antigens of pathogenic microorganisms, ensuring stable inheritance of the foreign genes (**Figure 1**). It has been confirmed that thymidine kinase (TK), gE, gI, and PK are closely associated with the virus’s virulence and neurotropism but do not affect its ability to replicate and express encoded antigens (23). This makes FHV-1 suitable for developing various pathogen vector vaccines. FHV-1 can establish latent infections in nerve cells, enabling a single administration of an FHV-1-based vector vaccine to potentially provide lifelong immunity (24). The virus exhibits high host specificity, being limited to feline species (25), with a low likelihood of cross-species infection. Furthermore, recombinant FHV-1 strains are easy to culture, can stably proliferate in F81/CRFK cells, and show clear cytopathic effects, facilitating the selection and purification of recombinant strains through plaque purification. Compared to other viral vaccines, FHV-1 has a lower lethality rate, providing high safety for vector vaccine development (23). Vaccines based on FHV-1 can be administered intranasally or via eye drops, making them convenient to use and easy to administer, with high market acceptance. These characteristics establish a solid foundation for developing FHV-1 as a vaccine vector.

## 3 Genetic manipulation techniques of feline herpesvirus

### 3.1 Homologous recombination

Homologous recombination (HR) involves the targeted insertion of exogenous genes into recipient cells. During replication, the homology between donor plasmid DNA and the flanking regions of the original DNA allows DNA exchange through HR, replacing the original gene with the exogenous DNA fragment (26). HR is fundamental for generating recombinant gene products. To construct recombinant feline herpesvirus, the target gene of the donor plasmid is typically flanked by homologous sequences at the breakpoints of the original gene. In early studies, HR was widely used in developing various viral vector vaccines (13–17). Recombinant FHV-1 constructed using this technique could stably inherit and express exogenous pathogenic genes, such as those from feline leukemia virus (27, 28) and feline immunodeficiency virus (29). However, HR efficiency is low, and the purification of recombinant strains requires complex screening processes, making experiments time-consuming and challenging (14). Since 2000, advancements in gene editing technologies have improved DNA cutting precision, reducing the difficulty of applying HR. This has yielded good results in developing viral vector vaccines, such as turkey herpesvirus expressing avian influenza virus hemagglutinin (30) and influenza virus expressing Helicobacter pylori neutrophil-activating protein (31).

### 3.2 Bacterial artificial chromosome

A bacterial artificial chromosome (BAC) is a bacterial chromosomal cloning vector constructed based on the F plasmid, capable of cloning DNA fragments up to approximately 150 kb. BACs offer high stability and a low likelihood of chimerism (32). The FHV-1 genome, about 136 kb in length, can be transformed into *Escherichia coli* using BAC, facilitating genetic modifications such as gene insertion or deletion. By appropriately modifying the vector, the viral gene-containing BAC can be transfected into eukaryotic cell lines for further recombinant virus modifications (33).

In 2006, scientists first attempted to create an FHV-1 BAC clone using the glycoprotein G gene as the insertion site (34). In 2010, another FHV-1 BAC clone was created with the upstream region of the UL56 gene as the insertion site (35). By inserting homologous regions flanking target sites into the FHV-1 BAC genome and co-transfecting it with purified DNA, multiple FHV-1 mutants were created. These mutants provide insights into developing attenuated FHV-1 vaccines (36). Establishing the BAC platform has significant value for *in vitro* cultivation of recombinant viral vectors, enhancing HR efficiency, and reducing the difficulty of constructing viral vectors. However, BAC sequences are challenging to remove, and the resulting recombinant viruses may carry unnecessary exogenous gene sequences, posing safety concerns (37).

### 3.3 CRISPR/Cas9 system

The CRISPR/Cas9 system is a mature gene-editing tool that uses nucleases to create double-strand breaks in the genome, providing insertion sites for target gene knock-ins and enhancing HR efficiency between the genome and linearized transfer vectors (38). Before CRISPR/Cas9, zinc finger nucleases (ZFNs) and transcription activator-like effector nucleases (TALENs) were the primary gene editing technologies. Both relied on nucleases to induce DNA repair processes, achieving gene editing through protein-guided DNA cutting (39). These methods required complex, time-consuming protein engineering, selection, and validation processes. The CRISPR/Cas9 system’s cutting specificity is guided by custom-designed guide RNA (sgRNA), allowing simultaneous targeting of multiple genes. It is simple to design, cost-effective, and highly efficient (40).

The first application of CRISPR/Cas9 in the herpesvirus genome was reported in 2015, demonstrating that adding CRISPR/Cas9 constructs during transfection/infection could increase the proportion of recombinant genomes post-transfection/infection, with recombinant gene products exceeding one-third (41). Specific modifications to FHV-1 using the CRISPR/Cas9 system can reduce the virus’s pathogenicity while maintaining immunogenicity, accelerating the screening and optimization of FHV-1 vector vaccine candidates. However, the CRISPR/Cas9 system’s non-specific delivery in tissue cells and potential base mismatches during sgRNA-DNA pairing can lead to erroneous Cas9 cleavage, causing DNA damage. Optimization methods such as sgRNA design, primer editing, and Cas9 nuclease modification aim to reduce off-target effects (42–44). Nonetheless, accurately and comprehensively detecting CRISPR/Cas9 off-target sites remains a significant challenge in gene editing.

## 4 Recombinant FHV as a vaccine vector expressing feline pathogens’ antigens

### 4.1 Feline leukemia virus

Feline leukemia virus (FeLV) belongs to the genus Gammaretrovirus in the Retroviridae family. FeLV infection can result in proliferative diseases (including lymphoma and myeloproliferative disorders), degenerative diseases (such as anemia and leukopenia), and immunosuppressive diseases associated with opportunistic infections (45). In 1990, FHV-1 was first used as a vaccine vector by constructing recombinant FHV-1 strains expressing the FeLV envelope (env) gene and the FeLV gag and gag protease genes, based on an FHV-1 TK gene deletion strain (27). Protein blot analysis of whole-cell extracts detected the expression levels of FeLV env precursor proteins (gp85 env precursor protein and gp70) and FeLV gag (**Table 1**).

**Table 1:** Summary of research on recombinant FHV-1 as a vaccine vector expressing antigens from other pathogens.

Pathogens	Target antigen(s)	Integration site	Immunization route	1^st^ immunization Dosage	Immunization interval/days	2^nd^ immunization dosage	Immune responses	Challenge infection	Protection	References
FeLV	env, gag	TK	/	/	/	/	/	/	/	(27)
	gp70, gp85, gag	TK	Intragastric, p.o.	1.3×10^5^ PFU/2×10^5^ PFU	27	1×10^7^ PFU/2×10^5^ PFU	/	8×10^6^ AcNPVgp85-infected St9 cells	Antibody titer rise	(28)
	env	ORF2	p.o., i.n.	10^4.7^ PFU, 0.75 ml	28	10^5.7^ PFU	NA titer was 1:32	10^5.6^ focus forming units (i.p.)	75% protection	(46)
FCV	capsid	TK	p.o., i.n.	1×10^6^ PFU, 1.0 ml	12	1×10^6^ PFU, 1.0 ml	NA titer was 1:40	/	/	(47)
	capsid	TK	p.o., i.n., intraocular	1×10^6^ PFU, 1.0 ml	/	/	NA titer was 1:4	1×10^8^ PFU, 1.0 ml (p.o., i.n., intraocular)	NA titer was increased 1:512	(48)
FPV	VP2	TK	s.c.	1×10^8^ TCID_50_, 1.0 ml	/	/	NA titer was 2^7.5^	1×10^6.5^ TCID_50_, 1.0 ml (p.o.)	100% protection	(49)
RABV	gG	gI/gE	i.n.	1×10^6.5^ TCID_50_, 1.0 ml	/	/	NA: 13.81 ± 8.29 IU/ml	10^4^ MIC LD_50_, 1.0 ml (masseter muscle)	100% protection	(50)
FIV	env	ORF2	s.c., p.o.	1×10^5^ PFU, 1.0 ml			NA titer was 1:16	10-20 CID50 (s.c.)	Antibody titer rise	(51)
	env	TK	/	/	/	/	/	/	/	(52)
	gag	TK	/	/	/	/	/	/	/	(53)
	env, gag	UL	p.o., i.n., s.c.	/	28	/	Not induce measurable immunity	/	No protection	(54)
	gag	TK	intraocular, p.o., i.n.	1×10^6^ PFU, 1.0 ml	/	/	Not induce antibody	/	/	(29)
*T. gondii*	SAG1	TK	/	/	/	/	/	/	/	(55)
	ROP2	TK	i.n.	1×10^6^ PFU, 1.0 ml	21	1×10^6^ PFU, 1.0 ml	IgG antibody	1×10^4^ bradyzoites (p.o.)	100% protection against cysts	(56)

□/, no information is specified;

NA, neutralizing antibody;

p.o., oral administration; i.n., intranasal immunization; s.c., subcutaneous injection.

In 1992, researchers improved this approach by inserting the gp70 gene between the human cytomegalovirus immediate early promoter and the BGH poly(A) signal, creating recombinant FHV-1 expressing the FeLV gp70 gene (28). Western blotting and immunoprecipitation of (3S) methionine-labeled proteins confirmed the expression of the gp70 protein in the recombinant strain. Further animal experiments assessed the protective effect of the recombinant FHV-FeLV vector vaccine against FeLV infection. Results showed that the gp70 protein was detectable in vaccinated cats, but the immune response was insufficient to provide adequate protection against FeLV infection (**Table 1**). The reason why vaccine immunity is inadequate is likely due to the inability of the local mucosal immune response to elicit a sufficient systemic immune response. Enhancing antigen expression levels and achieving comprehensive stimulation of systemic immune organs/systems to generate a robust immune response remains a challenge in FeLV vaccine development.

In 1996, researchers inserted the env gene downstream of the gC gene in the FHV-1 genome using homologous recombination and utilized the Rous sarcoma virus promoter to drive env gene transcription. The authors aimed to observe whether high-level env antigen expression could induce a stronger immune response. Compared to earlier studies, the recombinant FHV-1 stimulated high levels of neutralizing antibodies (VNA) (46), and provided some protection against FeLV infection, but not enough to fully protect experimental animals. Recombinant vaccines using canarypox virus as a vector to express FeLV env and gag antigens have shown to protect animals from FeLV challenge (57, 58), but it remains unproven whether the vaccine’s efficacy is due to cross-protection provided by multiple FeLV antigens (**Table 1**).

### 4.2 Feline calicivirus

Feline calicivirus (FCV) is a type of calicivirus that causes upper respiratory symptoms such as nasal discharge and coughing in felines (59). Since FHV-1 and FCV often co-infect cats and present similar symptoms, researchers have aimed to develop a combined prevention and treatment strategy for both viruses. In 1996, researchers used an FHV-1 TK deletion strain, inserting the capsid gene of the FCV F4 strain into the deletion site (47). Indirect immunofluorescence and immunoblotting verified the expression of FCV antigens in the recombinant FHV-1 strain. Animal vaccination experiments assessed the antibody response induced by the recombinant strain, demonstrating that the recombinant virus containing FCV antigens could induce specific neutralizing antibodies in the experimental animals (**Table 1**). However, this experiment did not evaluate the vaccine’s protective effect against FCV infection.

Yokoyama et al. enhanced recombinant antigen expression by adding an FHV-1 gC promoter sequence upstream of the inserted F4 FCV capsid gene (48). The resulting recombinant virus provided partial protection against FCV infection but did not achieve 100% efficacy (**Table 1**). Given that FCV is an RNA virus with a high mutation rate, vaccine development is particularly challenging. Selecting conserved FCV antigens and combining multiple antigens for immunization might offer a new direction for FCV vaccine development.

### 4.3 Feline parvovirus

Feline parvovirus (FPV), also known as feline panleukopenia virus, causes severe symptoms such as high fever, persistent vomiting, diarrhea, dehydration, and significant leukopenia. It is highly contagious and has a high mortality rate (60). Early clinical symptoms of FPV and FHV-1 infections are similar, and co-infections with these viruses are common, making dual prevention a key focus in feline vaccine development (4). Significant progress has been made in developing trivalent vaccines for FCV, FPV, and FHV-1. Zoetis in the United States has developed the Fel-O-Vax PCT inactivated vaccine, and Intervet in the Netherlands has developed the Nobivac Tricat Trio live attenuated vaccine, both capable of simultaneously preventing infections by FCV, FPV, and FHV-1. However, the presence of adjuvants, antibiotics, excipient proteins, or residual virulence in existing vaccines poses certain risks. Consequently, viral vector vaccines based on attenuated strains are gaining more attention due to their higher safety profile (61). A 2024 study reported a viral vector vaccine using an FHV-1 gIgE/TK virulence gene deletion recombinant virus expressing the FPV VP2 protein (49). Animal experiments showed that immunized cats exhibited milder clinical symptoms, fewer pathological changes, and reduced viral shedding post-infection. Additionally, the vaccine protected the animals from both FPV and FHV-1. The authors suggested that selecting immunogenic antigens, choosing appropriate insertion sites, and optimizing promoters could further enhance the vaccine’s immunogenicity.

### 4.4 Rabies virus

Rabies virus (RABV) is a single-stranded RNA virus in the Rhabdoviridae family that causes viral encephalitis in mammals and can be transmitted to humans through the saliva of infected animals. The prevention of RABV is closely related to public health safety (62, 63). In 2019, scientists constructed an FHV-1-based RABV candidate vaccine by combining the RABV gG gene with a CMV promoter, using an FHV-1 gI/gE gene deletion strain as the base (50). Immunized cats produced antibodies against both FHV-1 and RABV, showed no clinical symptoms of FHV-1 infection, and survived the challenge experiments (**Table 1**). These results demonstrated that the FHV-1-based recombinant vaccine could effectively protect against both FHV-1 and RABV infections, making it a potential candidate vaccine for RABV. However, since both RABV and FHV-1 can establish latent infections in the trigeminal ganglion, vaccinating animals with pre-existing immunity to RABV and/or FHV-1 could lead to an excessive viral load in the ganglia, potentially causing viral encephalitis. This study did not examine the latent infection of recombinant FHV-1 in the neurons of vaccinated cats, so the vaccine’s safety cannot be fully assured.

### 4.5 Feline immunodeficiency virus

Feline immunodeficiency virus (FIV) is an RNA virus belonging to the Retroviridae family. FIV infection leads to immunosuppression, making cats susceptible to a range of secondary infections, and is thus referred to as feline AIDS. Unlike human AIDS, feline AIDS is not transmitted through sexual contact but primarily through saliva during shared feeding and through scratches or bites, making group infections more likely (64). Currently, no drugs are available to treat FIV infection, and clinical treatment mainly involves symptomatic management, highlighting the importance of FIV prevention. In 1996, researchers first attempted to develop an FHV-1 vector vaccine against FIV by inserting the FIV env gene downstream of the gC homolog in the FHV-1 genome (51). Although the vaccine could express FIV antigens in vaccinated cats, the protection was inadequate, as all vaccinated cats showed symptoms of FIV infection (**Table 1**). Subsequent attempts included: (1) Inserting the full-length FIV env gene or a fusion of the FIV env gene with the FHV-1 gC promoter into the TK deletion site of the FHV-1 genome (52); (2) Inserting cDNA encoding the FIV gag protein into the TK deletion site of FHV-1 (53); (3) Linking the FIV gag gene with the FHV-1 gB promoter sequence and inserting it into the TK deletion site of FHV-1 (29); (4) Co-immunizing cats with recombinant FHV-1 expressing FIV env and recombinant FHV-1 expressing FIV gag genes (54). These approaches aimed to evaluate the feasibility of constructing an FIV vaccine using FHV-1 as a vector by assessing the expression levels of FIV proteins in vaccinated cats and the protective effects of the recombinant vaccine against FIV challenge. Although FIV antigens were detectable in vaccinated animals, none of the vaccines provided effective immune protection. This may be due to interference from pre-existing FHV-1 immunity affecting the immune response to FIV antigens. Researchers have suggested that targeting protective T-cell epitopes of FIV, rather than using the full-length env/gag genes, might offer better immunogenicity (65). Additionally, due to the instability of RNA viruses, developing vaccines effective against multiple FIV subtypes remains a key focus for preventing FIV infections.

### 4.6 Toxoplasma gondii

*Toxoplasma gondii* is a unicellular eukaryotic parasite capable of infecting almost all warm-blooded animals, posing a pathogenic risk to immunocompromised individuals (66). In 2001, researchers first constructed a recombinant FHV-1 expressing the *T. gondii* surface antigen (SAG1) (55). This recombinant FHV-1 (FHV/SAG1) demonstrated the potential of FHV-1 in T. gondii vaccine development by expressing the SAG1 antigen. In 2002, another recombinant FHV-1 expressing the *T. gondii* ROP2 antigen was developed (56). The recombinant FHV-1 (FHV/ROP2) induced an immune response, increasing serum IgG antibody levels, with effective antibody levels achieved after a booster immunization, blocking the formation of brain cysts (**Table 1**). However, due to post-translational modifications of protein targets and the multiple life stages of *T. gondii*, these recombinant FHV-1 vaccines could not reduce the production of T. gondii oocysts and did not provide full-stage immune protection. A cocktail protein vaccine developed using *T. gondii* calcium-dependent protein kinase 3 (CDPK3), dense granule protein 35 (GRA35), and *T. gondii* organelle protein 46 (ROP46) demonstrated that a complex immune stimulus from multiple *T. gondii* antigens could provide sufficient immune protection (67). Therefore, developing viral vector vaccines also needs to focus on combined immunization elicited by multiple *T. gondii* antigens. The unique advantage of FHV-1 as a vaccine vector lies in its genome containing multiple non-essential genes, allowing it to carry various *T. gondii* antigen genes, thus facilitating the development of *T. gondii* viral vector vaccines.

## 5 Challenges in developing FHV as a vaccine vector

### 5.1 Inconsistent standards for animal experiments

Animal experiments are indispensable in biomedical research and crucial for vaccine development. They allow for long-term, comprehensive observation of subjects, facilitating the evaluation of vaccine safety and immunogenicity. Therefore, the standardization (e.g., selection of experimental animals) and regulation (e.g., daily management of experimental animals) of these experiments are essential for ensuring the professionalism, reliability, and feasibility of the results (68). Currently, clinical trials of FHV-1 vaccines face three major issues: (1) Lack of Feline Model Animals: FHV-1 infection is species-specific, primarily affecting felines. Consequently, animal experiments for FHV-1 recombinant vaccines need to involve feline subjects. However, there are no established feline model animals internationally, restricting the clinical testing of FHV-1 recombinant vector vaccines. (2) Diverse Sources of Experimental Animals: There are no commercialized laboratory cats available domestically or internationally. Therefore, experimental animals for FHV-1 recombinant vaccine trials come from various sources, such as purchases from catteries, private breeders, or adopted stray cats. This variability among experimental animals leads to uncertainties in experimental results. (3) Challenges in Breeding Experimental Animals: Cats are naturally solitary animals that prefer a solitary and free lifestyle. They have high requirements for their living environment, including temperature, humidity, and space, making group breeding challenging and large-scale animal experiments difficult. These issues complicate the development of FHV-1 recombinant vaccines.

### 5.2 Preexisting immunity affecting vector vaccine efficacy

While there is ample theoretical basis for using feline herpesvirus as a vector to construct recombinant viral vaccines, no commercialized FHV-1 vector vaccines have been reported globally. The vaccines developed thus far have not achieved the protective efficacy expected of commercial vaccines. Various factors influence vaccine efficacy, one of which is the high prevalence of preexisting immunity to feline herpesvirus among felines. This preexisting immunity can limit the immune response to viral vector vaccines. Studies indicate that preexisting immunity models show a decline in the efficacy of related viral vector vaccines compared to immunologically naïve models (69, 70). FHV-1 is widespread globally, and a 2023 epidemiological survey of 1,158 cats with upper respiratory tract infections from 20 animal hospitals in Wuhan revealed a positive rate of 15.5% for FHV-1 (71). In the presence of preexisting immunity to FHV-1, using FHV-1 vector vaccines for immunization quickly activates the host’s specific humoral and cellular immune responses, leading to the rapid clearance or neutralization of FHV-1. Although some studies suggest that preexisting immunity does not significantly affect the efficacy of certain vaccines, this cannot be generalized to all related vaccines. Thus, addressing the immunosuppressive effects caused by preexisting immunity remains a critical focus in the development of FHV-1 vector vaccines.

## 6 Optimization of FHV as a vaccine vector

### 6.1 Optimization of the herpesvirus backbone

#### 6.1.1 Promoter optimization

Research suggests that expressing exogenous genes in tandem with promoter sequences yields better immunogenic effects compared to expressing single immunogenic exogenous genes. Moreover, the immune efficacy of the vaccine correlates with the level of exogenous gene expression in the viral vector (72). FHV-1 gene expression occurs in three phases: immediate-early, early, and late (73), with promoters regulating gene expression in a cascade manner during these phases. It is hypothesized that exogenous structural proteins in herpesvirus vectors are similarly regulated in a cascade fashion, with different promoters at various stages affecting exogenous gene expression differently. Promoters used in FHV-1 vector vaccine construction include endogenous viral promoters (gC, gD), the CMV promoter, and the Rous sarcoma virus (RSV) promoter. Studies have shown that the RSV promoter can induce exogenous gene expression levels nearly ten times higher than the CMV promoter (74). Selecting high-expression promoters is crucial for enhancing the immunogenic efficacy of FHV-1 vector vaccines. Additionally, heterologous promoters can reduce the immunosuppressive effects caused by preexisting immunity, further enhancing vaccine efficacy. Dual-promoter cascade regulation has been shown to induce higher expression levels than single-promoter systems (75). Although this technology has not yet been applied to FHV-1 vector vaccine construction, it offers new insights for optimizing these vaccines.

#### 6.1.2 Codon optimization

Codon optimization involves adjusting the synonymous codons of inserted genes according to the host’s codon preferences to enhance recombinant protein expression, thereby improving vaccine immunogenicity (76). This strategy has been validated for enhancing viral vector vaccine efficacy (77). Different species have distinct codon preferences, and herpesviruses have unique codon optimization requirements. Current strategies primarily focus on enhancing the immunogenicity of monoclonal antibodies, such as using OptimWiz to increase the expression of the gD gene of herpesvirus, thereby enhancing the immunogenicity of the gD protein (78). Codon optimization can also be used as a virulence attenuation strategy, known as codon pair bias deoptimization (79), which aids in developing attenuated vaccines and improves the safety of viral vector vaccines.

#### 6.1.3 Knockout of viral virulence or immune evasion genes

Genetic modifications, such as knocking out virulence-related genes and those involved in immune evasion, can significantly enhance the safety and immunogenicity of vaccine vectors. While the immune evasion mechanisms of FHV-1 are not fully understood, the well-characterized mechanisms in herpes simplex virus (HSV) offer valuable insights. HSV employs various strategies to antagonize the immune system. For instance, the HSV protein ICP47 inhibits antigen presentation by MHC class I molecule (80, 81), and ICP34.5 counteracts the antiviral effects of RNA-dependent protein kinase (PKR) (82). The Us3 protein can suppress the immune killing function of CTL cells (83). Research on herpes simplex virus type 1 (HSV-1) has shown that knocking out genes related to immune evasion can enhance the immunogenicity of the viral vector, thereby improving the immune efficacy of the virus-based vaccine. For example, the HSV-1 multiple immediate early gene d106 deletion mutant can induce high levels of sustained foreign gene expression while reducing the virus’s virulence (84). Among the various virulence factors of HSV, ICP34.5, a neurotoxic factor encoded by late genes, is closely associated with viral replication and pathogenicity, making it a focal point of research. Studies targeting ICP34.5 in HSV vector vaccines have demonstrated that knocking out ICP34.5 can significantly reduce the neurotoxicity of HSV while retaining immunogenicity. Additionally, further deletion of ICP47 can optimize the oncolytic properties of HSV, paving the way for its application in cancer vaccine research (85).

In addition, the immune response of the host to the viral vector itself can diminish the intensity of immune stimulation triggered by the foreign antigen carried by the vector. This effect is further exacerbated in the presence of preexisting immunity, which can significantly reduce the overall immune protection provided by the vaccine. Modifications to the viral vector capsids can help mitigate this issue. For example, equipping vector capsids with peptides that interfere with toll-like receptor signaling can diminish the innate and adaptive immune responses induced by the capsids (86). Furthermore, these capsid modifications have the potential to minimize the negative impact of preexisting immunity, thereby enhancing the effectiveness of the vaccine vector (87).

### 6.2 Optimization of the virus backbone carrying exogenous genes

The herpesvirus genome can carry a larger number of foreign genes compared to adenoviruses and adeno-associated viruses, allowing for the addition of foreign cytokines and chemokines to potentially enhance both humoral and cellular immunity. For example, a study on a herpes simplex virus-related vaccine demonstrated that recombinant HSV-1 expressing interleukin-4 (IL-4) significantly reduced the pathogenicity of the recombinant HSV vaccine and increased the survival rate of immunized mice after HSV challenge (88). This result highlights the potential for simultaneously inserting foreign antigen genes and cytokines into the herpesvirus genome to boost the immunogenicity of antigen vaccines. The strategy of enhancing vaccine immunity through the incorporation of foreign cytokines and chemokines has been extensively explored in the development of HSV-related DNA vaccines. Studies have shown that cytokines like IL-12, IL-15, IL-18, and IL-21 can synergize with HSV antigens to reduce the pathogenicity of HSV while maintaining the immunogenicity of HSV proteins (89). This approach offers a promising pathway for developing safer and more effective viral vector vaccines.

## 7 Perspective

Since the 20th century, research on FHV-1 vector vaccines has advanced rapidly. The stability of FHV-1 and its species-specific infection characteristics provide a solid theoretical foundation for using FHV-1 as a vaccine vector. Techniques such as homologous recombination and CRISPR/Cas9 offer robust support for the swift and stable development of recombinant FHV-1 vector vaccines. Substantial research has accumulated on using feline herpesvirus as a vaccine vector, demonstrating some protective effects against FHV-1 and related viral infections post-immunization. However, their immunogenicity does not yet meet the levels required for herd immunity vaccines. Utilizing molecular adjuvants and heterologous promoters to enhance immunogenicity could provide new avenues for FHV-1 vector vaccine development. Additionally, using mucosal viral vector vaccines to induce site-specific immune responses might significantly enhance protection against mucosal pathogens (72), such as FHV-1 vector vaccines expressing FCV and FPV antigens. This hypothesis requires validation through extensive clinical trial data.

FHV-1, a linear double-stranded DNA virus, can encode approximately 74 proteins. Some proteins, like glycoproteins gB, gD, gH, and gL, are essential for viral replication and cannot serve as insertion sites for exogenous genes. However, non-essential genes for viral replication, such as gC, gE, gG, and gI, can be used as insertion sites. Studies on several α-herpesviruses have demonstrated that recombinant vector vaccines constructed using TK, gI, and gE insertion sites can maintain the immunogenicity of exogenous genes (90, 91). However, the efficacy of gC and gG sites remains unverified. As research on FHV-1 vector vaccines progresses, developing trivalent, tetravalent, and even pentavalent vaccines will likely become a focal point. Consequently, exploring related protein functions should also be a research priority. Furthermore, studies on recombinant pseudorabies virus (RPV) have shown varying safety and protection levels among different gene deletion vaccines. For instance, TK/gE/gI triple deletion recombinant PRV is safer than gE/gI double deletion mutants, and gI/gE/TK/UL13 quadruple deletion strains offer higher protection and safety compared to gI/gE/TK deletions (92, 93). These findings have yet to be confirmed in recombinant FHV-1 vector vaccines but comparing the safety and protection of various FHV-1 deletion strains could provide substantial support for breakthrough research in vector vaccines.

Although significant progress has been made in FHV-1 vector vaccine research, current vaccines do not yet meet the desired immunogenicity standards. Future research on feline herpesvirus vector vaccines should focus on further attenuating FHV-1 virulence and screening relevant immunogenic antigens. Comprehensive studies on the *in vitro* characteristics of candidate FHV-1 vaccines and the *in vivo* immune mechanisms of FHV-1 will aid in advancing candidate vaccine research. Applying new technologies such as reverse genetics, bioinformatics, molecular biology, and immunology can provide theoretical and technical support for the immunogenicity research of the feline herpesvirus vector vaccine platform.
